# Exploring adherence to the International Confederation of Midwives’ Essential Competencies in Europe

**DOI:** 10.18332/ejm/224435

**Published:** 2026-07-09

**Authors:** Chiara Valentini, Laura Batinelli, Lucia Rocca-Ihenacho, Daniela Drandic

**Affiliations:** 1 Nursing and Midwifery Department, City St George's University of London, London, United Kingdom; 2 International Confederation of Midwives, The Hague, the Netherlands

**Keywords:** Europe, professional identity, midwife-led care, ICM essential competencies, midwifery autonomy

## Abstract

**INTRODUCTION:**

Midwives play a critical role in improving maternal and neonatal outcomes, with midwifery-led care proven to reduce unnecessary interventions and empower women. Yet, across Europe, systemic and cultural barriers continue to hinder midwives’ ability to fully adhere to global standards such as the International Confederation of Midwives (ICM) essential competencies.

**METHODS:**

A qualitative study captured the perspectives of ten midwives from five European countries: UK, Germany, Belgium, Bulgaria, and Italy. Purposive sampling captured diverse representation from midwives working within national regulatory frameworks and practicing independently. Semi-structured interviews provided rich insights into essential competencies adherence, with thematic analysis revealing key trends.

**RESULTS:**

Participants identified shared challenges, including societal undervaluation of the midwifery profession, workforce strain, and restrictive legislations, alongside regional differences including governance barriers and lack of regulation of out-of-hospital births. Benefits to adherence to ICM competencies included enhanced autonomy and opportunities for international collaboration. Overarching themes were structural and cultural as external dynamics, and professional as internal dynamics. The former included medicalization, subordinate role of midwives, and regulatory barriers, whereas at a professional level emerged relationship-centered care and theory-practice gap, as key subthemes. Proposed strategies included strengthening adherence, fostering academic and institutional support, and enhancing professional standing.

**CONCLUSIONS:**

This study highlights that essential competencies are not fully implemented across Europe. Urgent collaboration among academic and health institutions, as well as midwifery associations, is essential to create enabling environments and strengthen essential competencies’ adherence in Europe. Ultimately to fostering professional identity and improving maternal and neonatal health outcomes.

## INTRODUCTION

Midwives can prevent up to 4.3 million maternal and neonatal deaths annually by 2025, according to ‘The Midwifery Accelerator’^1,2^. Scaling up the midwifery workforce and increasing access to midwifery-led care (MLC) are recognized strategies to reduce maternal and neonatal mortality and morbidity^2-4^.

As primary maternity care providers, midwives are uniquely positioned within healthcare systems (HSs) to support the mother–baby dyad and address the dual challenge of ‘too much too soon’ and ‘too little too late’, referring to both overuse of unnecessary medical interventions and delays in escalation of care when clinically indicated^2,5,6^. Over-medicalization is linked to higher rates of birth trauma, reduced maternal satisfaction, and long-term physical and psychological consequences, increased healthcare costs, and impact on future mode of birth^2,6-8^. In contrast, MLC emphasizes physiological processes, personalized care, and timely access to emergency services, and is associated with improved outcomes, women’s satisfaction, empowerment, and cost-effectiveness^2,4,6,8,9^.

Despite strong evidence of their impact^10^, shortages of midwives persist globally and across Europe^1-4^. Realizing midwives’ full potential requires an ‘enabling environment’ characterized by respect within multidisciplinary teams, strong professional identity, adequate resources, and a sense of belonging^1,10-13^. The term ‘professional identity’ encompasses values, skills, and sense of belonging that shape how a profession sees itself, central to practice and workforce sustainability^14^. Without addressing constraints on the scope of practice, progress toward maternal and neonatal health targets remains limited^2-4^.

In Europe, midwifery is a regulated profession with a defined scope of practice shaped by national education and regulatory frameworks^15^. The ICM is a non-governmental organization representing National Midwifery Associations (NMAs) in over 110 countries and promoting global standards for education, regulation, and practice^16^. The ICM essential competencies for midwifery practice outline the knowledge, skills, and professional conduct required for high-quality, evidence-based practice (EBP)^17^. Following a major review in 2024, the essential competencies underwent substantial changes not only from four to five domains, but in competencies, reflecting current and innovative EBP and MLC^17,18^. Increased focus on sexual and reproductive health and rights, physiological birth, technology, and gender-based violence. The five domains now covered are: cross-functional competencies; sexual and reproductive health and rights; antenatal care; care during labor and birth; and ongoing care of women and newborns^17,18^.

Together with the EU Directive 2013/55/EU (updating Directive 2005/36/EC), which ensures comparability of qualifications and professional mobility across member states^19^, these frameworks ensure safe and consistent maternity care across Europe. However, the Directive has seen minimal change since 1980, remaining largely outdated and misaligned with current international standards, creating inconsistencies in education standards and quality maternity care within Europe^19,20^. Recent efforts to revise the Directive offer a timely opportunity to address these gaps and better align European midwifery regulation with ICM essential competencies^19,20^.

As a result of these inconsistencies, adherence to the ICM essential competencies varies considerably across Europe^21^. Differences in HS organization, governance, professional recognition, cultural attitudes toward childbirth, and resource availability shape how midwives are trained, valued, and able to practice^5,21,22^. Midwives frequently report limited autonomy, professional marginalization, and restricted access to continuing professional development (CPD), particularly within hierarchical and medically dominated systems^4,15,22,23^. Cultural norms and professional power dynamics influence collaboration within multidisciplinary teams, often positioning midwifery as subordinate and undermining confidence in physiological childbirth^4,23^. Under-resourced HSs further constrain care aligned with international frameworks and standards^2,5,10,12,21,22^. Recognition of qualifications and professional autonomy also varies; while some countries grant midwives independence, others impose restrictive scopes of practice and limited CPD access, contributing to fragmentation within the profession^11,15,22^.

The diversity of European HSs governance, funding, and service organization shapes the availability of MLC models and the extent of midwifery autonomy^5,15^. While some countries enable practice aligned with ICM essential competencies across settings, others restrict midwives’ practice largely to hospital-based roles^1,11^. This interplay of structural, cultural, and professional factors creates barriers to consistent implementation of global frameworks, such as ICM essential competencies, scope of practice, and EU Directive^4,5^.

Existing research has examined midwifery education, regulation, and professionalization globally^24^ or within single European contexts^15,22^, as well as specific clinical themes, such as mode of birth, or competency adherence assessed through simulated scenarios^25,26^. However, qualitative research on the implementation of the ICM essential competencies across Europe and in diverse professional contexts remains limited. Addressing this gap, the present study offers an initial cross-country qualitative exploration of adherence to ICM essential competencies, identifying shared barriers and regional variations. By amplifying midwives’ voices, it provides insights into how education, policy and practice may better align with international standards, such as ICM essential competencies, and lay the groundwork for future comparative research.

Specifically, the aim of this study is to explore midwives’ perspectives on adherence to the ICM essential competencies, including those working within NMAs and those practicing independently, across the five European regions: Northern, Southern, Eastern, Central and Western Europe.

## METHODS

### Study design and sampling

An empirical qualitative study design was employed to explore how midwives across Europe interpret and apply the ICM essential competencies. The study was informed by an interpretivist epistemological position, which understands knowledge as socially constructed and meaning as co-constructed between researcher and participants, rather than as an objective, fixed truth^27^. A qualitative approach was considered appropriate to capture in-depth perspectives on professional practice, autonomy, and contextual influences shaping the implementation of ICM essential competencies within diverse HS settings.

Participants were recruited using purposive sampling to ensure context-specific experience relevant to the research aims^28^. Recruitment occurred primarily through the Midwifery Unit Network (MUNet) group, a European community of practice, and through professional networks of the research team. This strategy enabled access to midwives with direct experience of applying the ICM essential competencies in different educational, regulatory, and work contexts. Sampling was guided by two criteria: geographical representation and professional diversity. One country was selected from each of the five European regions (Northern, Western, Central, Eastern, and Southern Europe). From each country, two midwives were recruited, one midwife affiliated with an NMA and one independent midwife (IM).

Countries were selected based on feasibility of recruitment and guidance from the state of the world’s midwifery report^4,29^. Inclusion criteria were qualified midwives, NMA-affiliated or IM, familiar with the ICM essential competencies prior of the interview, and fluency in English or Italian. Exclusion criteria were unqualified or unregistered midwives, midwives outside selected regions, affiliation to the ICM, and those not fluent in English or Italian.

### Data collection process

Data were collected through semi-structured, one-to-one online interviews conducted between July and October 2024, following ethical approval. Semi-structured interviews were chosen to allow flexibility while ensuring coverage of key topics, including understanding and application of the ICM essential competencies in individual contexts, perceived barriers and facilitators to this adherence, professional autonomy, and system-level influences^30^. All participants received detailed written information about the study and provided informed consent before participation, with the right to withdraw at any stage without consequence. Prior to the interviews, participants received an information sheet summarizing outlining the study aims and summary of the ICM essential competencies, along with a link to the full document, to ensure familiarity with the framework.

Interviews lasted approximately 60 minutes and were conducted via telecall. An interview guide was developed in English and Italian to support linguistic inclusivity and depth of expression. The guide was used flexibly, allowing participants to elaborate on issues of relevance to their context and experience. All interviews were recorded with participants’ consent and transcribed verbatim. Transcripts were checked against recordings for accuracy.

### Data analysis and interpretation

Data were analyzed using reflexive thematic analysis following Braun and Clarke’s six-step approach: familiarization with the data, initial coding, theme development, review, and definition, and reporting^31^. This method was selected for its structured but flexible analytic process to identify shared meanings across diverse contexts while preserving sensitivity to individual perspectives. All transcripts were imported into NVivo qualitative data analysis software to support systematic coding and organization. Initial coding was conducted inductively, with codes generated directly from the transcripts. These codes were then reviewed and grouped into subthemes and overarching themes through iterative comparison across cases and regions, with attention to both convergence and variation. The resulting themes revealed the contextual, professional, and systemic influences on adherence to essential competencies for midwifery practice.

Reflexivity was embedded throughout the research process to ensure that interpretations remained grounded in participants’ accounts rather than professional assumptions^32^. The researcher’s professional background as a midwife supported an in-depth understanding of midwifery practice, professional language, and contextual realities, facilitating rapport and rich, meaningful dialogue. Collaborative and reflective practices included the use of a standardized interview guide, participant validation of responses, anonymized coding, peer debriefing with co-authors, and reflexive journaling following each interview^32^. An audit trail was maintained to document analytic choices and reflexive insights throughout the process. Where required, translation support was used to preserve cultural and professional meaning. Acknowledging the researcher’s position fostered transparency and ethical integrity, supporting the authentic representation of diverse midwifery perspectives.

## RESULTS

Ten midwives participated in the study, 2 for each European region: United Kingdom (Northern), Germany (Central), Belgium (Western), Bulgaria (Eastern), and Italy (Southern Europe). NMA representatives contributed policy-oriented perspectives, while IMs offered pragmatic views on everyday practice and regulatory gaps. Notably, 2 participants identified as educators alongside their NMA affiliation (UK and Germany), which may limit the depth of insight into educational contexts; this is acknowledged as a limitation of the study. Participants ranged in the ages from 25 to ≥54 years, with experience from <10 to >40 years, representing academic institutions, NMAs and diverse work settings such as hospitals and community, private practices. Following the interview guide, all participants identified key benefits, challenges, and strategies related to adherence to ICM essential competencies within their contexts. Despite differences in regulation and practice, participants shared recurring experiences of professional undervaluation and subordination within hierarchical systems. Both IMs and NMA representatives agreed that stronger adherence to ICM core competencies could benefit professional autonomy, standing, and job satisfaction. A shared strategy across groups was the need to strengthen professional connections, counteracting isolation and inspiring confidence to step beyond subordinate roles. Benefits cited included promoting EBP, improving care quality, and expanding women’s options. Bulgarian participants highlighted adherence to reinforce EBP and guideline use, while Belgian and German midwives mentioned improved continuity of care and intra-European knowledge exchange. Distinct barriers emerged between groups. NMAs focused on regulatory constraints, while IMs faced more immediate priorities, including workforce shortages and poor collaboration. Overall, NMAs prioritized scaling up the profession through legal frameworks and political engagement, whereas IMs favored grassroots strategies like advancing relationship-centered care (RCC) and expanding academic opportunities and curricula, as suggested by Bulgarian midwives. In Italy, both NMA and IM midwives presented unified perspectives: they viewed enhanced professional status as the main benefit of adherence and undervaluation as the greatest barrier. Both groups strongly advocated for the regulation of out-of-hospital births as a key strategy to promote midwives’ autonomy and adherence to ICM essential competencies. In the following findings, competency numbers refer to the relevant ICM essential competencies for midwifery practice, illustrating where real-world practice diverges from global standards.

**Figure 1:**
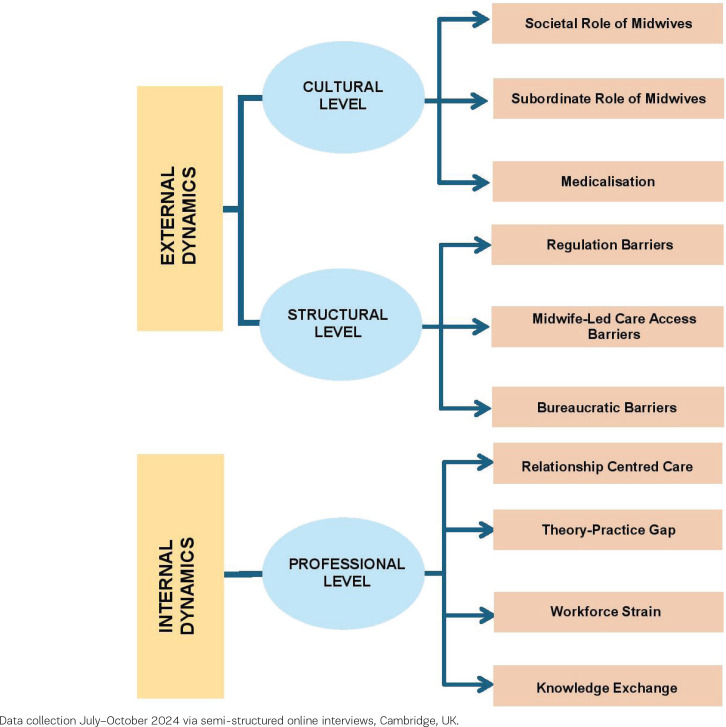
Themes and subthemes graphic in exploring ICM competencies in Europe, an empirical qualitative study, 2025 (N=10)

These findings reveal patterns across Europe, conceptualized in three overarching themes: external cultural dynamics, external structural dynamics, and internal professional dynamics, each influencing adherence to ICM essential competencies (Figure 1).

### External dynamics: cultural level

Cultural norms shape how midwifery and midwives are viewed, respected, and integrated within HSs and society overall.

*Societal role of midwives*

Across all countries, participants described cultural norms that devalue midwifery, undermining autonomy and public recognition. In the UK, inquiries and media coverage often highlight failings rather than successes, eroding trust. In Italy and Belgium, midwives are often equated with nurses, obscuring their distinct role. In Germany, obstetricians’ dominance further marginalizes midwives to the public, while in Bulgaria, independent midwifery was described as ‘exotic’:

*‘So independent midwives are not very common in Bulgaria. It’s something rare yet. And let’s say exotic for now. So, the people are not accepting it very well.’* (IM, Bulgaria)

*Subordinate role of midwives*

Cultural undervaluation intersects with systemic hierarchies, reinforcing midwives’ subordinate status within HSs. In Italy, systemic expectations pressure midwives to defer to doctors, while in Germany, restrictive insurance policies and obstetric dominance diminish autonomy. Bulgarian participants described stark power imbalances, noting:

*‘Doctors are not required to have midwives, but midwives are required to work with doctors. … We do not receive the lobbying that doctors do, because they are all in Parliament and other policymaking structures.’* (NMA midwife, Bulgaria)

*Medicalization*

Within these hierarchical structures, models of care are also affected, so that obstetric-led models dominate, hindering midwives’ ability to provide physiological care, a central topic of the core competencies. Participants from Italy and the UK described defensive practices driven by fear of litigation:

*‘Both midwives and the medical staff, are often driven by fear of malpractice or legal complaints and this leads to defensive medicine. To me, this is very sad; seeing that society is moving further away from respecting what constitutes a good birth and instead becoming even more medicalized.’* (NMA midwife, Italy)

In these systems, midwives are part of a large, industrialized system that can disconnect them from the core competencies of midwifery. Examples included the absence of one-to-one care in Germany (contravening Competency 4.a), routine supine positions in Belgium (4.b), and persistently high cesarean rates in Italy and Bulgaria. Bulgarian midwives also noted that even basic practices like skin-to-skin contact and exclusive breastfeeding were rarely supported (4.b, 5.c). Medicalization also affects midwifery education, as mentioned by the NMA Italian midwife, which is led by medical professionals. This fosters a mindset prioritizing pathology over physiology, undermining midwives’ confidence and adherence to core competencies.

### External dynamics: structural level

While cultural attitudes shape the perception of midwifery, structural barriers, laws, governance, and HSs design define what midwives can actually do.

*Regulation barriers*

Regulatory frameworks across countries were noted to be either inadequate or absent. In the UK, the removal of statutory home-visit requirements weakened postnatal care. In Belgium, poor reimbursement policies limit midwifery services, while in Italy and Bulgaria, out-of-hospital births remain either unregulated or illegal, exposing midwives to legal risks (1.n, 3.g):

*‘You are not allowed to attend a birth without participation of a doctor. And if you go to home birth and something happened, you are not safe at all. So, there are no contract, nothing regulated. It’s grey and tricky area.’* (IM, Bulgaria)

Governance issues exacerbate these challenges. German NMAs lack regulatory authority, while in Belgium, the absence of mandatory membership limits advocacy and access to CPD.

*MLC access barriers*

Except in the UK, where MLC is integrated within the national HS, access elsewhere is fragmented and inequitable. Geographical disparities, labeled as a ‘postcode lottery’ by a UK NMA midwife, further exacerbate inequalities. In Germany, where in some areas MLC is not widely available compared to others, or in Italy, where the south experiences higher rates of medicalization compared to more privileged northern regions. In the UK and Belgium, continuity of care can vary significantly depending on regional leadership and local policies. In Bulgaria, women often relocate temporarily to access MLC:

*‘There are two midwifery practices for the whole country in the capital. And that’s why a lot of people are coming (to the capital) to give birth, taking Airbnbs or some other ways to be there for the last month of the pregnancy and to have a chance for natural delivery.’* (IM, Bulgaria)

*Bureaucratic barriers*:

Across the UK, Italy, and Belgium, bureaucratic and administrative demands were consistently described as constraining holistic and relationship-centered care (RCC), with documentation and procedural compliance reducing time for meaningful interaction and personalized support:

*‘Midwives are very busy. They’re doing a lot. They’re doing this form and they’re doing this checklist. But are they really looking at women holistically and really listening to what they want?’* (NMA midwife, UK)

In Italy, IM reported delays with emergency services during out-of-hospital births, undermining timely collaboration. In Belgian hospitals, the rigid scheduling, time-keeping practices, and institutional performance pressures prioritize efficiency over relational continuity.

### Internal dynamics: professional level

Internal professional factors, such as education, philosophy of care, working conditions, and knowledge exchange, also shaped adherence to ICM essential competencies.

*Relationship-centered care*

Participants identified RCC and trust as the foundation of MLC, improving outcomes and experiences:

*‘If I ever must change a hairdresser, there’s no way I’d want a different hairdresser every time. And that’s just hair, that’ll grow. … When I had my children, I wanted to know who was caring for me, to feel that they trusted and had confidence in me. I managed that as a midwife, but not every woman can navigate or achieve that.’* (NMA midwife, UK)

Bulgarian NMA midwife remarked RCC as cost-effective and beneficial for both women and HSs. Independent practice was seen as better supporting RCC than hospital settings across Italy, the UK, Belgium, and Germany:

*‘The one-to-one continuity of care works very well within the realm of independent freelance midwives but not so well in the hospital settings, or not at all.’* (IM, Germany)

*Theory-practice gap*

A theory-practice gap was evident across most countries. Bulgarian midwives cited outdated curricula, while Italian midwives sought additional training at personal expenses to meet global standards, and UK midwives described learning about autonomy and physiological care not matching the reality of the workplace:

*‘Midwives are trained to be autonomous practitioners … once they get out into practice, that is no longer what they’re experiencing. So, there is very little trust in physiology. There is too much too soon, too little too late. That culture is terrifying.’* (IM, UK)

This gap undermines Competency 1.a ‘Assume responsibility for own decisions and actions as an autonomous practitioner’.

*Workforce strain*

Staff shortages, burnout, and poor work-life balance were highlighted by all participants, directly affecting compliance with ICM core competencies:

*‘Newly qualified [midwives] don’t want to work full-time anymore. They want a better life balance.’* (NMA midwife, UK)

Midwives described feeling unable to provide the quality of care they aspired to, expressing frustration that institutional expectations and pressures set a minimum threshold rather than encouraging holistic and RCC:

*‘The system in hospital, you don’t have to do more, but I wanted to do more.’* (IM, Belgium)

*Knowledge exchange*

Limited opportunities for international collaboration and CPD hindered adherence to Competencies 1.b and 1.e. Bulgarian, Belgian and German participants highlighted the lack of Erasmus and EU-level training programs. Professional learning and exposure to diverse practices were seen as key to strengthening professional identity and motivation:

*‘The only way we have to understand who we are and where we want to go is to study, stay informed, and keep doing so.’* (NMA midwife, Italy)

Across all interviews, a unified thought emerged: investing in MLC is essential. Despite clear evidence of proven benefits, midwives remain undervalued. This concerning paradox underscores the urgent need for a shift in policy, as well as changes in societal and professional attitudes toward midwifery.

## DISCUSSION

This study confirms and extends existing research, revealing diverse applications of ICM essential competencies across Europe that limit midwives’ capacity to practice to their full scope^20,33^, and in non-enabling environments for the application of the essential competencies^1,12^.

### Cultural level

Cultural perceptions strongly influenced how midwives are valued within HSs and society. Misconceptions about midwives’ roles dilute professional identity and autonomy, reducing demand for MLC, despite clear advantages of this model of care^2,5,14,22^. Participants described entrenched hierarchies and over-medicalization across all countries, confirming pre-existing literature^2,15,22,33^. Feminist critiques trace this marginalization to patriarchal norms privileging male-dominated obstetrics over the female-led domain of midwifery^23^. Such hierarchies corrode authority, devalue physiology, and restrict women’s choices^23,33^. Consistent with other European studies^15^, participants across all five countries described institutional hierarchies that marginalize midwives, suggesting these dynamics are systemic rather than country-specific.

### Structural level

Fragmented governance of NMAs emerged as a barrier to awareness and implementation of ICM essential competencies^13,15,21^. The EU Directive ensures the minimum education and practice standards for midwifery recognition and practice; however, regulatory inconsistencies persist among countries^19-21^. Belgium lacks mandatory NMA membership, Germany’s NMA has no legal authority, and Bulgaria’s is minimally recognized. These gaps weaken advocacy and limit midwives’ policy influence, contrary to global recommendations emphasizing strong NMAs to advance the profession^13^. Recent efforts to revise the EU Directive represent an important opportunity to align European regulatory standards with current ICM essential competencies and WHO guidelines. Updating minimum education and practice requirements could help address the regulatory fragmentation identified in this study, strengthen NMA authority, and support more consistent, high-quality maternity care across Europe^20^. Consequently, the poor integration of midwife-led services within HSs further reflects systemic neglect for physiological care and women’s reproductive autonomy^4,5,34^.

### Professional level

A recurring theme was the lack of a professional voice, which weakened advocacy for MLC and diminished belonging^14^. Professional isolation and stagnation reflect broader international trends constraining midwives’ roles. These limitations challenge adherence and reduce the capacity to deliver safe, woman-centered care, which are core values of midwifery^2,4,10^. Strengthening respect, empowerment, and belonging, key elements of an enabling environment, is essential for improving maternal and neonatal health^1,4,11,13^. Educational gaps, including insufficient CPD and international exchange, perpetuate stagnation^1,4,11^. Weak representation in academic and healthcare decision-making bodies widens the gap between global frameworks and national realities^13,15^. Participants viewed essential competencies as an opportunity to foster shared professional identity, qualification comparability, and professional mobility across Europe, aligning with ICM and EU strategies^18,20^. Adherence to ICM essential competencies could support cross-border collaboration and collective advocacy^20^.

### Opportunities for progress

Participants highlighted the transformative potential of ICM essential competencies in strengthening professional identity, recognition, and belonging^4,21^. Policy alignment with ICM essential competencies, investment in leadership, CPD, and midwife-led research are urgently needed to bridge the theory-practice gap and dismantle systemic barriers^2,4,13,20,22^. Advancing midwifery in Europe, therefore, requires coordinated action among educational and regulatory bodies and policymakers. The NMAs should lead standardization and advocacy, EU institutions must ensure up-to-date directives aligned with international standards, such as ICM essential competencies, and national governments must ensure adequate staffing, resources, and working conditions^2,4,12,20^. Academic institutions should embed ICM essential competencies in curricula and promote cross-border exchange. Future research should adopt longitudinal, mixed-methods designs with larger samples to evaluate the impact of reforms and strategies to shift societal attitudes.

### Strengths and limitations

This study offers insights into the realities of ICM essential competencies’ implementation across Europe, with participants from all five regions capturing diverse cultural, structural, and regulatory dimensions. Participation of both NMA representatives and IMs provided institutional and grassroots insights. Semi-structured interviews enabled flexibility, depth, and reflexivity. Thematic analysis using NVivo enhanced transparency and structure. Limitations include the small sample size and time constraints, restricting broader contextual exploration. Purposive sampling may have attracted particularly engaged participants. Additionally, only 2 participants identified as educators through academic affiliations, which may limit the depth of insight into educational contexts specifically; future research would benefit from greater educator representation.

## CONCLUSIONS

This study identified cultural, structural, and professional barriers to adherence to ICM essential competencies across Europe. Entrenched hierarchies, fragmented governance, inadequate regulation, and limited professional identity consistently undermine midwives’ autonomy and scope of practice. Participants identified policy alignment with ICM essential competencies, strengthened professional networks, and cross-border collaboration as key strategies to advance midwifery practice and consolidate professional identity across Europe. Coordinated action across educational, regulatory, and institutional levels is urgently needed to close the gap between global standards and national realities and deliver consistent, high-quality maternal and neonatal care.

## Data Availability

The data supporting this research are available from the authors on reasonable request.
